# ENVOLVER+: a tool for the promotion of positive nursing practice environments

**DOI:** 10.3389/fpubh.2025.1488660

**Published:** 2025-02-12

**Authors:** Olga Maria Pimenta Lopes Ribeiro, Ligia Schacht, Mariana Filipa Mendes Gonçalves, Ana da Conceição Alves Faria, Marlene Patrícia Ribeiro, João Miguel Almeida Ventura-Silva

**Affiliations:** ^1^Nursing School of Porto, Porto, Portugal; ^2^Center for Health Technology and Services Research (CINTESIS@RISE), Porto, Portugal; ^3^Santa Catarina State University, Florianopolis, Brazil; ^4^ICBAS, Abel Salazar Institute of Biomedical Sciences, University of Porto, Porto, Portugal; ^5^Médio Ave Local Health Unit, Santo Tirso, Portugal; ^6^Tâmega and Sousa Local Health Unit, Penafiel, Portugal; ^7^Portuguese Red Cross Northern School of Health, Oliveira de Azeméis, Portugal

**Keywords:** board game, game, nursing, professional practice, working environment

## Abstract

**Introduction:**

Patients, nurses, and the organization itself can benefit from a good work environment. One of the weaknesses most often cited by nurses was the lack of involvement and participation in setting workplace policies. Using games is a promising strategy for promoting positive caregiving environments. The purpose of this study is to describe the process by which the ‘ENVOLVER+’ board game has been developed and validated.

**Methods:**

We carried out a methodological study from September 2023 to May 2024 in three phases: (1) modified e-Delphi to validate the content to be included in the game; (2) development of the prototype board game; (3) application of the game in four practice contexts.

**Results:**

The four sessions of the ‘ENVOLVER+’ game lasted an average of 80 min. Each session had twelve participants in four groups. At the end of the sessions, we asked the participants to comment on the advantages and disadvantages of the game.

**Discussion:**

The ‘ENVOLVER+’ game can be a valuable tool in promoting a positive nursing practice environment by providing an innovative and interactive approach to ensuring nurse involvement and participation.

## 1 Introduction

From the perspective of the International Council of Nurses (ICN), a positive environment for nursing practice ensures decent and excellent work, promotes nurses’ safety, health and well-being, quality of care, motivation, productivity, and individual and organizational performance (1). They are characterized by innovative structures and policies that focus on the recruitment and retention of professionals, continuous training and professional development strategies, appropriate compensation systems, recognition programs, adequate resources, and a safe working environment (1).

However, this was not and is not the reality of many nurses’ clinical practice contexts, which is why in 2007, the ICN celebrated International Nurses’ Day with the theme ‘Positive Practice Environments: Quality Workplaces = Quality Patient Care’ to raise awareness of the need to create positive practice environments for nurses (1). In 2023, the ICN reiterated the same intention. It added the need to ensure adequate remuneration and the ability to retain professionals (2). In addition to the ICN’s goal, other organizations, such as the World Health Organization (WHO) and the International Labor Organization (ILO), have taken positions on the need to improve working environments. Examples include the publication of the report ‘The State of the World’s Nursing 2020: Investing in Education, Jobs, and Leadership’ by WHO, which provides a comprehensive analysis of the state of nursing and makes recommendations for improving the working environment for nurses; and the inclusion of safe and healthy working conditions in the ILO framework on fundamental principles and rights at work (3, 4).

In its report on International Nurses’ Day 2024, the ICN emphasizes that the health sector, and nursing in particular, is crucial to the economic and social development of countries (5). The issue is that, due to an aging population, changing disease patterns with an increase in chronic diseases, and growing expectations of health care services, a significant shortage of nurses is expected in the coming years, drawing even more attention to this field (4, 5). After the initial enthusiasm and celebration of the ‘frontline heroes’ during the pandemic, there is now renewed discussion of high workloads, staff shortages, burnout, and low salaries (5).

To create decent working conditions that attract and retain nurses, the ICN has identified five priority fields: (1) investing in education and nurses by funding nursing education and creating jobs; (2) improving working conditions, including work-life balance, safe practice environments, and opportunities for professional development; (3) valuing nurses’ work, including fair pay, recognition of their role, decent working conditions, and a focus on nurses’ well-being; (4) promoting gender equality in the workplace, reducing gender inequalities, and creating more equitable opportunities; and (5) investing in collaboration between the health sector and other sectors to maximize the economic benefits of investing in nursing (5).

In line with what is defined by the ICN, research conducted in recent years in pre-pandemic, pandemic, and post-pandemic contexts has provided relevant data on the working conditions of nurses and their impact (6–12). The results of these and other studies show that a supportive environment for nursing practice has positive effects on nurses, with higher levels of job satisfaction and engagement, better perceptions of the quality of care provided, lower levels of burnout, and lower intentions to leave the profession; on patients, with lower mortality rates and higher levels of satisfaction; and on organizations, with lower turnover and absenteeism, less omitted care, and improved quality of care (13–19). Because of this triple effect, investment in this field has been recommended internationally (20, 21). In Portugal, research has shown that, in addition to low pay and lack of recognition, nurses’ involvement and participation in the policies, strategies, and functioning of the institution and/or service in which they work are some of the main weaknesses (9, 16, 22–24). Additionally, within the scope of the concept analysis of a positive nursing practice environment, the involvement and participation of nurses were considered fundamental contributions to improving work environments (25).

Literature reviews identify interventions, practices, and educational programs that have been implemented to improve nursing practice environments (26–28). Regarding how interventions are implemented to improve nursing practice environments in hospital settings, Paguio et al. (28) categorized them into three approaches: accreditation processes, educational strategies, and a participatory approach.

Interventions designed to achieve a set of criteria required by an accrediting body are categorized as accreditation process strategies (28). Interventions focused on educational strategies, conducted through training, lectures, and workshops, aim to improve nurses’ competencies, including procedural performance, communication skills, and leadership (28). Finally, in the participatory approach, interventions aim to implement innovations in units, improve components of the nursing process, and promote leadership, teamwork, autonomy, and communication (28).

The authors concluded that the interventions associated with the participatory approach were the ones that most consistently showed positive effects on nurses, patients, and organizations (28). However, the literature has identified the need for further studies to develop and test the effectiveness of interventions implemented to improve nursing practice environments to strengthen existing knowledge and provide models that can be replicated and incorporated into organizational policy (19, 28).

The COVID-19 pandemic has led to unimaginable advances in innovation and technology, creating new opportunities for health interventions. The need to develop strategies to optimize the efforts of professionals has accelerated, and serious games are one possible solution that has received considerable attention in the literature (29).

In recent research, gamification and, particularly, serious games in education within the context of health professions have emerged as transformative educational approaches that not only enhance learning and retention but also develop other essential competencies for healthcare professionals (30). From the authors’ perspective, these games have the potential to improve knowledge and skills, fostering decision-making, teamwork, and communication (30).

Although the use of games in healthcare has been widely recognized, the focus is often centered on the development of clinical knowledge and skills (31), leaving a gap regarding their use in interventions aimed at improving professional environments.

Considering the above and the need to invest in participatory approach interventions within the scope of practice environments, as part of a broader project on nursing practice environments, this study aims to describe the development and validation process of the board game ‘ENVOLVER+’.

## 2 Materials and methods

We carried out a methodological study (32) for the development and validation of the ‘ENVOLVER+’ board game in three phases: (1) modified e-Delphi to validate the content to be included in the game; (2) development of the prototype board game; and (3) application of the game in four practice contexts.

### 2.1 Study design

To validate the game content, we conducted a modified e-Delphi study in Phase I. The Scale for Environment Evaluation of Professional Nursing Practice (SEE-Nursing Practice) (20, 33), developed as part of the larger Positive Professional Environments for Nursing Practice (PPE4NursingPractice) project, the literature review, and previous studies (9, 16, 22, 23), served as the basis for the initial game content. Given the diversity of sources, the Delphi technique was essential to validating the content included in the game. After two rounds, we reached unanimity among the experts. To ensure rigor, we conducted this study according to the Recommendations for the Conducting and Reporting of Delphi Studies (CREDES) (34).

In Phase II, we developed the prototype of the board game after deciding what would be included in the game. This phase included creating the design, creating the logo, and choosing the name ‘ENVOLVER+’. The design includes a component to encourage reflection and discussion on pertinent practice environment issues, as well as another component to validate knowledge on the same issues, since one of the main goals is to encourage nurses’ involvement in promoting positive nursing practice environments.

Phase III involved testing the game in four practice contexts in a hospital in northern Portugal. Following the implementation of the game, we asked participants to provide input on the advantages and disadvantages of this technology.

### 2.2 Participants

In Phase I, we used a non-probabilistic purposive sampling technique to select the experts. In this study, ‘experts’ are professionals with extensive training and experience in the field of nursing practice environments. The specified requirements for inclusion were as follows: (a) having worked as a nurse for at least five years; (b) having expertise or involvement in research initiatives related to nursing practice environments; and (c) being willing and able to participate in all required Delphi rounds. Although there is no consensus regarding the ideal number of experts, following the guidelines of Niederberger and Spranger (35) and considering the risk of dropout across different rounds, 20 experts were selected. We contacted participants by e-mail, which included an official invitation letter and a link to access the study. Upon accessing the study, we informed the participants of the objectives, purpose, potential benefits, and informed consent, which they had to accept before starting the questionnaire. Anonymity and confidentiality were guaranteed, and all participants were informed that they could withdraw from the study at any time without repercussions.

In Phase II, researchers developed the game prototype based on modified e-Delphi results. To encourage player interaction, we chose a board game with a jigsaw-like component and cards, thus fulfilling one of the main goals of the game: to encourage the participation of all nurses in promoting positive nursing practice environments.

In Phase III, we implemented the game in four practice settings in a hospital in the northern region of Portugal, following an expression of interest from the respective nurse managers. A total of 48 nurses—12 from each practice setting where the game was used—participated in this test phase using a non-probabilistic convenience sampling technique.

### 2.3 Data collection and analysis

In Phase I, when the modified e-Delphi was implemented, it was based on the premise that the consensus reached by a group is more reliable than individual points of view. The experts rated the content of the game using a four-point Likert scale: (1) strongly disagree; (2) somewhat disagree; (3) agree; and (4) strongly agree.

We organized the expert validation data, and then we calculated the Content Validity Index (CVI): the number of responses 3 and 4 divided by the total number of responses (32). Based on this calculation, we revised and reformulated the content with a CVI < 0.90. The CVI must be equal to or greater than 0.90 (32).

#### 2.3.1 First round

We developed the questionnaire using Microsoft Forms, and it had two sections: the first consisted of sociodemographic and professional characterization, and the second presented questions related to the game, namely the rules of the game, the content of the 59 REFLETIR+ cards, and the 59 SABER+ cards. We asked the experts to suggest improvements for content rated as ‘strongly disagree’ or ‘somewhat disagree’. At the end of the questionnaire, all experts had the opportunity to make suggestions. We emphasize that the questionnaire was pre-tested with four nurses and four researchers who met the inclusion criteria and were not involved in this study, which made it possible to assess the comprehensibility of the questions asked.

We analyzed the content of each card to make changes based on CVI and expert advice.

#### 2.3.2 Second round

After incorporating feedback from the first round, we sent a revised version of the game content to the experts in the second round. As in the first round, all cards were validated to assess stability. As in the first round, at the end of the questionnaire, we asked all the experts to make suggestions. We collected and analyzed the scores and comments to determine the content validation performed by the experts.

We used descriptive statistics to analyze the sociodemographic and professional characteristics of the participants and to calculate the CVI.

In phase III, after applying the game in the four practice contexts, a two-part Microsoft Forms-created questionnaire was sent to the 48 nurses who had taken part in the dynamic. In the first part, the sociodemographic and professional characterization, and in the second part, two open-ended questions, one on the advantages and the other on the disadvantages of using the game. Descriptive statistics were used to analyze the sociodemographic and professional characteristics of the 48 participants. The three stages of the operational proposal for content analysis were followed in the study of the participant’s responses to the open-ended questions: pre-analysis (the stage of arranging the data that comprised the research corpus), exploration of the content, and inference and interpretation (36).

### 2.4 Ethical considerations

The development and validation of the ‘ENVOLVER+’ board game are components of a broader research project that has received ethics committee and hospital approval (process no. 104/21). It is important to remember that all participants signed an informed consent form, and that the data collected was anonymized and kept confidential. To maintain anonymity for the purposes of the qualitative findings, each response was coded with the initial letter of the word participant, followed by the number.

## 3 Results

The results are displayed based on the three phases: (1) Modified e-Delphi to validate the content to be included in the game; (2) Development of the prototype board game; (3) Application of the game in four practice contexts.

### 3.1 Phase I – Validation of game content

We constructed the content included in the game based on a literature review, previous studies (9, 16, 22, 23), and the Scale for Environment Evaluation of Professional Nursing Practice (SEE-Nursing Practice) (20, 33).

We contacted twenty experts inviting them to participate in the first round of the modified e-Delphi. Ninety percent of the participants completed the questionnaire after agreeing to participate. Only one expert did not respond in the second round within the allotted time. The sociodemographic and professional details of the experts who participated in the two rounds of the modified e-Delphi are shown in Table 1.

**Table 1:** Sociodemographic and professional characterization of the experts.

Sociodemographic and professional characteristics	First round (*n* = 18)	Second round (*n* = 17)
Gender *n* (%)		
Female	12 (66.7)	12 (70.6)
Male	6 (33.3)	5 (29.4)
Marital status *n* (%)		
Single	4 (22.2)	4 (23.5)
Married/non-marital partnership	14 (77.8)	13 (76.5)
Age (years) mean; std. dev.	39.2; ± 6.3	39.1; ± 6.5
Education *n* (%)		
Bachelor’s degree	4 (22.2)	4 (23.5)
Master’s degree	9 (50.0)	9 (53.0)
Doctoral degree	5 (27.8)	4 (23.5)
Job title *n* (%)		
Nurse	3 (16.7)	3 (17.6)
Nurse specialist	9 (50.0)	9 (53.0)
Nurse manager	2 (11.1)	1 (5.9)
Professor	4 (22.2)	4 (23.5)
Time of professional practice (years) mean; std. dev.	17.2; ±6.5	17.1; ±6.7

Source: authors. std. dev. standard deviation.

In the first round, each expert accepted the name and logo of the game. Ninety-five percent of the participants agreed with the rules of the game. We calculated the CVI for the content contained in the 59 cards (REFLETIR+ cards) to encourage reflection and discussion on relevant topics in the practice setting. Based on the content validation, we individually revised and adapted the cards with a CVI less than 0.90. Based on expert recommendations we reworded six cards. We conducted the second round after considering the improvement of ideas.

The CVI improved because of the six cards changes; it was higher than 0.90 for each card (Table 2).

**Table 2:** Validation of the content of REFLETIR+ Cards by experts.

REFLETIR + Cards	First round	Second round	REFLETIR + Cards	First round	Second round
	CVI	CVI		CVI	CVI
1	**0.88**	**0.94**	31	**0.88**	**0.94**
2	1.00	1.00	32	1.00	1.00
3	1.00	1.00	33	1.00	1.00
4	1.00	1.00	34	1.00	1.00
5	1.00	1.00	35	1.00	1.00
6	1.00	1.00	36	1.00	1.00
7	1.00	1.00	37	**0.94**	**1.00**
8	1.00	1.00	38	1.00	1.00
9	1.00	1.00	39	1.00	1.00
10	1.00	1.00	40	1.00	1.00
11	**0.88**	**1.00**	41	1.00	1.00
12	1.00	1.00	42	1.00	1.00
13	1.00	1.00	43	1.00	1.00
14	1.00	1.00	44	1.00	1.00
15	1.00	1.00	45	1.00	1.00
16	1.00	1.00	46	1.00	1.00
17	**0.88**	**0.94**	47	1.00	1.00
18	1.00	1.00	48	1.00	1.00
19	1.00	1.00	49	1.00	1.00
20	1.00	1.00	50	1.00	1.00
21	1.00	1.00	51	1.00	1.00
22	1.00	1.00	52	1.00	1.00
23	1.00	1.00	53	1.00	1.00
24	1.00	1.00	54	**0.88**	**1.00**
25	1.00	1.00	55	1.00	1.00
26	1.00	1.00	56	1.00	1.00
27	1.00	1.00	57	1.00	1.00
28	1.00	1.00	58	1.00	1.00
29	1.00	1.00	59	1.00	1.00
30	1.00	1.00			

Source: authors. CVI, content validity index. Values in bold correspond to the CVI of cards whose content has been subjected to two rounds.

We calculated the CVI for the content of the 59 cards designed to validate knowledge of relevant topics in practice settings (SABER+ cards). Based on the validation of the content, we revised and adapted the cards that individually received a CVI less than 0.90. Based on the experts’ suggestions, we improved the wording of 18 cards. After incorporating the suggestions for improvement, the second round took place. The changes made to the 18 cards resulted in an improvement in CVI that was greater than 0.90 for all cards (Table 3).

**Table 3:** Validation of the content of SABER+ Cards by experts.

SABER + Cards	First round	Second round	SABER + Cards	First round	Second round
	CVI	CVI		CVI	CVI
1	1.00	1.00	31	**0.83**	**0.94**
2	1.00	1.00	32	1.00	1.00
3	**0.78**	**0.94**	33	**0.88**	**1.00**
4	1.00	1.00	34	1.00	1.00
5	**0.94**	**1.00**	35	1.00	1.00
6	1.00	1.00	36	**0.88**	**1.00**
7	1.00	1.00	37	1.00	1.00
8	1.00	1.00	38	1.00	1.00
9	**0.88**	**1.00**	39	**0.94**	**1.00**
10	1.00	1.00	40	1.00	1.00
11	**0.83**	**0.94**	41	1.00	1.00
12	1.00	1.00	42	**0.94**	**1.00**
13	1.00	1.00	43	1.00	1.00
14	1.00	1.00	44	1.00	1.00
15	1.00	1.00	45	**0.94**	**1.00**
16	1.00	1.00	46	1.00	1.00
17	**0.94**	**1.00**	47	**0.88**	**1.00**
18	1.00	1.00	48	**0.88**	**1.00**
19	1.00	1.00	49	1.00	1.00
20	**0.88**	**1.00**	50	1.00	1.00
21	**0.88**	**0.94**	51	1.00	1.00
22	1.00	1.00	52	1.00	1.00
23	1.00	1.00	53	1.00	1.00
24	1.00	1.00	54	**0.83**	**1.00**
25	1.00	1.00	55	1.00	1.00
26	**0.88**	**1.00**	56	1.00	1.00
27	1.00	1.00	57	1.00	1.00
28	1.00	1.00	58	1.00	1.00
29	**0.94**	**1.00**	59	1.00	1.00
30	1.00	1.00			

Source: authors. CVI—content validity index. Values in bold correspond to the CVI of cards whose content has been subjected to two rounds.

The first phase of the modified e-Delphi, which involved an expert panel, enabled the content of the 59 ‘REFLETIR+’ and 59 ‘SABER+’ cards to be validated. The first fifty-nine cards, named REFLETIR+, are categorized into three dimensions: Structure (29 blue REFLETIR+ cards), Process (19 purple REFLETIR+ cards), and Outcome (11 green REFLETIR+ cards). These cards relate to the subjects covered in SEE-Nursing Practice. The remaining 59 cards allude to the SABER+ component, which consists of 59 pertinent subjects with questions covering a range of nursing practice settings. The SABER+ cards are also arranged in three dimensions: Structure (29 blue SABER+ cards), Process (19 purple SABER+ cards), and Outcome (11 green SABER+ cards) based on the references utilized.

### 3.2 Phase II – Development of the board game prototype

**Figure 1:**
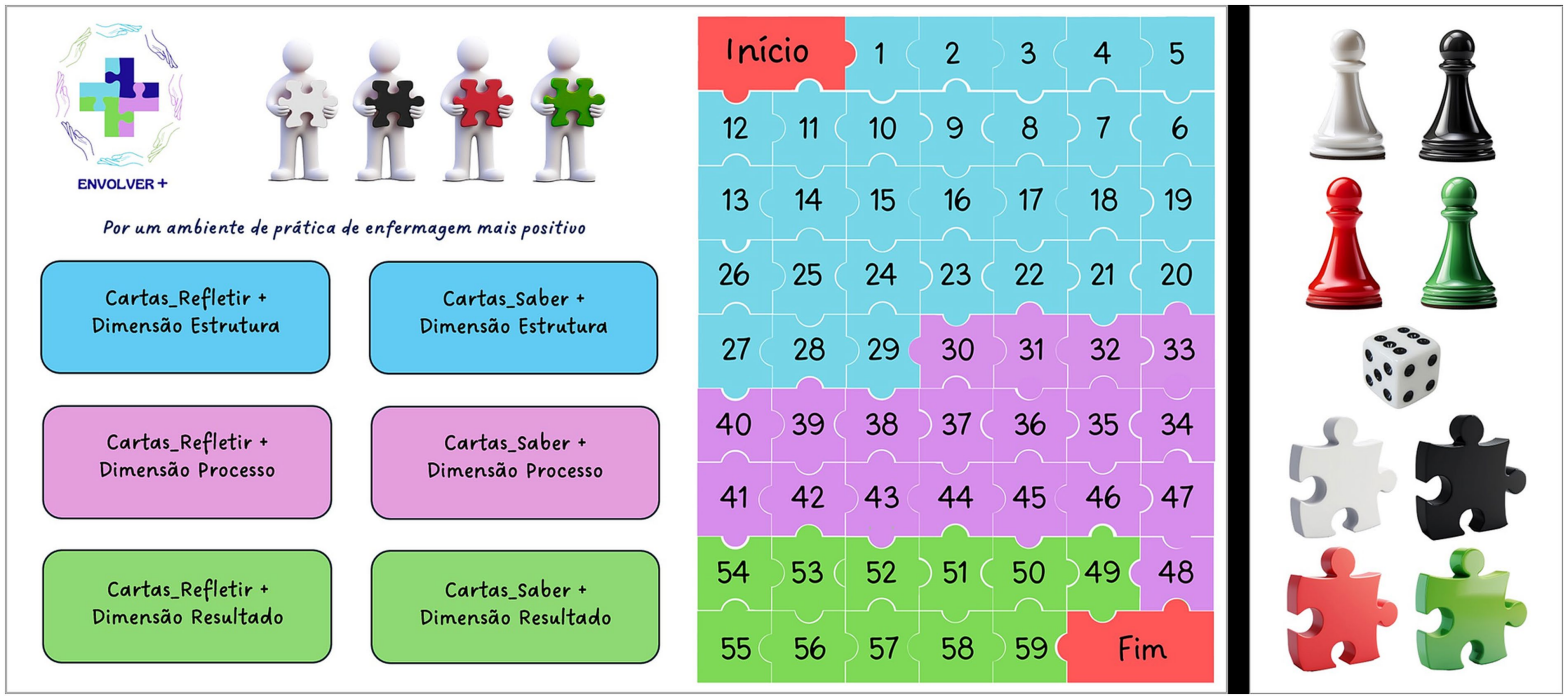
Prototype game “ENVOLVER+”.

In the prototype game called ‘ENVOLVER+’ depicted in Figure 1, the board on the right contains a jigsaw puzzle, with one piece marking the beginning. Fifty-nine game pieces, ending with one piece marking the end; 59 REFLETIR+ cards (with a specific space on the board); 59 SABER+ cards (with a specific space on the board); 4 pawns; 1 dice; and 4 loose jigsaw pieces, colored white, black, red, and green (to be allotted to each team).

Apart from the prototype shown, the rules of the game stipulate that the number of players must be between eight and twenty-four, divided into four groups. The game begins with each player on the piece marked ‘Start’. The objective of the game is to move the pawn around the 59 puzzle pieces according to the number rolled on the dice. A player on one of the teams is aware of the theme, which is shown from four angles, when he stops on a particular ‘piece’.

The four participating teams are required to engage in the REFLETIR+ component based on the color of the puzzle piece assigned to them. The role of the white puzzle team is to present objective facts and information; the role of the black puzzle team is to present negative risks and aspects; the role of the red puzzle team is to address emotions and approach the issue; and the role of the green puzzle team is to present innovative and hopeful improvement strategies. After this period of reflection and discussion, the SABER+ component asks a question on the same topic. Each team’s goal in the game is to assemble four puzzle pieces to form the word APE+ (Ambiente de Prática de Enfermagem Positivo), which stands for ‘Positive Nursing Practice Environment’.

### 3.3 Phase III – Application of the board game

The third phase of ‘ENVOLVER+’ game application sessions lasted an average of eighty minutes and were conducted in four hospital units. Each session was attended by twelve nurses, divided into four groups. At the end of the session, participants completed a questionnaire about the advantages and disadvantages of the game.

The sociodemographic and professional characteristics of the 48 nurses who participated in this phase of the study are shown in Table 4.

**Table 4:** Sociodemographic and professional characterization of participants.

Sociodemographic and professional characteristics	
Gender *n* (%)	
Female	32 (66.7)
Male	16 (33.3)
Marital status *n* (%)	
Married/non-marital partnership	33 (68.7)
Single	13 (27.1)
Divorced	2 (4.2)
Age (years) mean; std. dev.	38.2; ± 8.2
Education *n* (%)	
Bachelor’s degree	41 (85.4)
Master’s degree	7 (14.6)
Job title *n* (%)	
Nurse	35 (72.9)
Nurse specialist	13 (27.1)
Time of professional practice (years) mean; std. dev.	15.3; ± 8.7

Source: authors. std. dev. standard deviation.

Content analysis of the 48 participants’ comments resulted in the six subcategories displayed in Table 5 under the heading ‘Benefits of using the game’. The most frequently mentioned benefits by the participants are education about the characteristics of the practice environment, stimulation of team spirit, and everyone’s involvement.

**Table 5:** Subcategories and registration units within the “advantages of using the game” category.

Subcategories	*n*	Registration units: examples
Competence in the dimensions of the practice environment	10	“…it’s a way of getting to know the environment in which you work…” (P6) “…knowing all the fields to invest in, increases the likelihood of making a significant contribution to a more positive environment…” (P13)
Strategy for continuous improvement of the practice environment	7	“…using the game, we can clearly see that there is always the possibility to improve something…” (P21) “…using the game to stimulate joint reflection with a view to continuous improvement is fantastic…” (P36)
Involve everyone	16	“…it is an excellent strategy to ensure that everyone is involved…” (P31) “…the game addresses one of the main complaints of the nurses: the lack of participation in decisions…”(P8)
Problem-solving facilitator	6	“…the fact that teams look at problems from four different perspectives makes problem-solving more effective…” (P7) “…by analyzing the problem, looking at the facts, the negative aspects, what you feel and what could be done to improve…we have more and better information…which contributes to better problem solving…” (P35)
Foster team spirit	12	“…the use of the game promotes a sense of belonging to the team and a desire to contribute to the common good…” (P40) “…the fact that the game encourages joint reflection/discussion stimulates team spirit…” (P43)
Team motivation	9	“…with this dynamic, the team is motivated to do more and better…” (P39) “…taking into account everyone’s opinion is a motivating aspect for the team…” (P22)

Source: authors.

Regarding the category ‘Disadvantages of using the game’, Table 6 shows the four subcategories that emerged from the responses of the twelve players. The most frequently mentioned disadvantages were the time required to maximize the dynamics and the requirement of a minimum number of participants.

**Table 6:** Subcategories and registration units within the ‘disadvantages of using the game’ category.

Subcategories	*n*	Registration units: examples
A minimum number of participants is required.	5	“…I understand the importance of needing at least 8 participants, but this aspect can be an obstacle…” (P9) “…the minimum number of participants requires prior booking of the activity where the game will be played … because we do not have 8 nurses per shift” (P11)
Time is needed to make the most of the dynamics.	7	“…the game has a lot of potential; the hard part is finding the time to use it and make the most of it…” (P12) “…the reflection/discussion generated by the game requires time, which is always limited. …” (P46)
Readiness to use the game frequently	3	“…to work, the team has to show a willingness to use the game as a strategy…” (P2) “…sometimes there is a tendency to hold quick meetings without much reflection/discussion…in fact, colleagues have to be willing to stick to the strategy…” (P17)
Team members are not identifying with the strategy	3	“…there’s always a colleague who does not identify with this strategy…that you think will not work…” (P18) “…there are colleagues who do not value these strategies, they think it’s a waste of time…” (P33)

Source: authors.

## 4 Discussion

This three-phase study’s objective was to provide an overview of the creation and validation of the board game ‘ENVOLVER+’. The online format made it easier to quickly access experts and insights, and the modified e-Delphi proved to be a suitable technique for content validation. The experts’ comments and low dropout rate from the first to the second round demonstrate their interest in and dedication to the topic, despite the length of the content that needs to be evaluated.

Although the content of the SABER+ cards was more extensive and involved a greater workload for the experts in terms of validation and suggestions for improvement, the results obtained contributed to the qualification of the tool. The rapidity of the experts’ responses allowed the researchers to incorporate all the suggestions into the game prototype, reinforcing the relevance of serious games designed with the participation of experts in the validation of design, rules, and content (37).

The results of Phase III make it clear that the ‘ENVOLVER+’ board game has the potential to achieve one of the primary goals of its development: to increase nurses’ involvement in promoting positive nursing practice environments.

Using the REFLETIR+ cards, nurses can reflect together on each nursing environment topic from four perspectives: facts and information, risks and negatives, emotions, and strategies for improvement.

In addition, this tool has the potential to increase nurses’ knowledge of the dimensions of the nursing environment, particularly through the SABER+ cards.

In addition to enhancing nurses’ understanding of the nursing environment, the game is proving to be an important tool in planning and implementing change where interaction and teamwork are essential (9). When using the game, it became clear that the involvement of everyone and the stimulation of team spirit were the most frequently mentioned benefits by the nurses.

By promoting nurses’ engagement and knowledge of the dimensions and components of the nursing practice environment, this initiative has the potential to contribute to creating decent working conditions, reducing inequalities, and promoting fair and safe practice environments for nurses, which is effectively aligned with the United Nations Sustainable Development Goals (SDGs) (38). This alignment is notable in relation to SDG 8, which aims to promote decent work for all; SDG 10, which aims to reduce inequalities and combat gender discrimination; and SDG 16, which aims to promote safe working environments and protect workers’ rights and seeks to build effective, accountable, and inclusive institutions. In addition, the motivation generated by the greater involvement of nurses in using the game also ensures alignment with SDG 3, which aims to ensure that health workers are prepared and motivated to provide quality care (5, 38).

The ‘ENVOLVER+’ game, a strategy for improving care environments using a participatory approach (28), is also aligned with the Quadruple Aim, which focuses on four goals: (1) improve the experience of care recipients; (2) improve the experience of caregivers; (3) improve health outcomes; and (4) reduce health care costs (39). In fact, initiatives to improve the environment for nursing practice will have the triple impact on patients, nurses, and organizations already described in the literature.

Our goals for this tool influenced our decision to use a board game rather than a digital game. Board games are typically played in groups, but digital games, especially video games, are typically played alone. According to Martinez et al. (40), there is a current trend toward the use of online video games where players can interact, but these interactions are virtual, as opposed to board games where players primarily interact socially and physically. We chose this board game because the most important element of ‘ENVOLVER+’ is the interaction between players—in this case, nurses.

The benefits of involving nurses in decision-making in healthcare institutions have been highlighted by several authors (9, 10), so the use of games can be a compelling strategy to allow nurses to express their opinions and influence the decisions that affect their daily lives.

In recent years, the COVID-19 pandemic has led to rapid changes in the learning and working environment (23). Methods and techniques that do not attract attention are ineffective. In addition, the entry into the workforce of Generation Z, which has been described as the generation born and raised with innovation and technology (41), has supported the idea that games may be particularly promising for educational and work contexts. This has attracted interest in academia and practice, with several authors mentioning the increased motivation and participation of those involved (29, 42, 43).

In addition to the difficulties posed by their work environment, nurses are part of a professional community that also experiences emotional upheaval from the pressures of their demanding workloads, responding to patients’ needs, and attending to their own needs for self-care, well-being, and professional growth (16, 44). These encounters typically lead to elevated stress levels, which in turn lead to burnout and a breach of professional well-being. Improving nurses’ well-being has a major impact on the standard of care, but consistent and sufficient organizational support is needed for these professionals to perform well and be more resilient.

In this context, collaboration with other stakeholders, such as team colleagues, service managers, and the institutions themselves, is fundamental to creating a positive environment for nursing practice, and it is undeniable that a good workplace has the potential to contribute not only to the professional well-being of nurses but also to patient and organizational benefits.

Regarding the role of leaders in promoting nurse involvement, authors (45) warn of the importance of nurse managers being authentic with their subordinates and adopting strategies that ensure their participation. This can lead to a healthier work environment and a relationship of trust between the nursing team and their leader, which often results in greater satisfaction among professionals, greater reflection on their practice, and improved care (22).

Chiao and Niu (43) assert that games are a powerful and significant type of strategy. In this case, nurse managers have a viable solution to the problems that regularly arise in the context of professional practice: the ‘ENVOLVER+’ game.

### 4.1 Implications for practice

Feedback from participants in ‘ENVOLVER+’ game implementation sessions indicates that this tool enhances nurses’ problem-solving, communication, and ability to deal with a variety of workplace issues, in addition to encouraging their involvement and participation. This approach can lead to a more positive nursing practice environment for all involved by encouraging innovative thinking, which can also increase motivation and improve professional skills. The implications for nursing education and clinical practice are far-reaching.

Through the REFLETIR+ and SABER+ cards, the game encourages comprehensive reflections and contributes to deepening knowledge about the work environment. The game dynamics promote active participation, addressing one of the main challenges reported by nurses: the lack of involvement in institutional decision-making. Additionally, it strengthens team spirit and collaboration, fostering a sense of belonging and collective commitment.

Another strength of the game is its ability to facilitate problem-solving by providing a structured approach that considers multiple perspectives and fosters constructive discussions. The ‘ENVOLVER+’ also stands out as a motivational strategy, sparking interest in continuous improvements and aligning with the Sustainable Development Goals. The positive impact of this tool has the potential to be felt by healthcare professionals, patients, and organizations, making it a valuable and replicable solution.

### 4.2 Limitations

Despite the rigor with which the phases of the study were conducted, there are some limitations. First, the modified e-Delphi involved a purposefully selected panel of experts, which may have excluded potential experts in the field. The use of the game in four practice contexts in the same institution, with the participation of 48 nurses identified through a convenience sampling strategy, requires a cautious interpretation of the results and calls for validation of the game in other contexts. Although it was not the objective of this study, it is suggested to conduct pre- and post-intervention research as well as comparative studies to evaluate the effectiveness and benefits of the ‘ENVOLVER+’ game in promoting positive nursing practice environments.

## 5 Conclusion

The ‘ENVOLVER+’ game includes two components: one focused on reflection and discussion on the Nursing Practice Environment dimensions (REFLETIR+ cards), and the other focused on promoting literacy on the Nursing Practice Environment dimensions (SABER+ cards). Expert feedback and the experience of implementation in four practice contexts were essential in qualifying the tool.

The results of this study suggest that ‘ENVOLVER+’ can be a valuable tool in promoting positive nursing practice environments by providing an innovative and interactive approach to nurse involvement and participation. This study also makes a valuable contribution by addressing the potential of board games in qualifying nursing practice environments. This tool allows us to explore ways in which nurses can actively participate in policymaking and engage in initiatives that positively influence the professional practice environment.

The use of the ‘ENVOLVER+’ game will have an impact not only on health care professionals, but also on patients and health care organizations.

## Data Availability

The original contributions presented in the study are included in the article, further inquiries can be directed to the corresponding author.
